# Lessons from Reverse Translation

**DOI:** 10.1371/journal.ppat.1005516

**Published:** 2016-06-16

**Authors:** Adam S. Lauring

**Affiliations:** 1 Division of Infectious Diseases, Department of Internal Medicine, University of Michigan, Ann Arbor, Michigan, United States of America; 2 Department of Microbiology and Immunology, University of Michigan, Ann Arbor, Michigan, United States of America; The Fox Chase Cancer Center, UNITED STATES

Like many scientists, I sometimes stumble when faced with the cocktail party question—“So what exactly do you study?” After muttering something along the lines of, “basic mechanisms of virus evolution,” the discussion often moves on to the safer ground of politics or religion. As a physician-scientist, I often feel an extra layer of guilt in describing my work, as though I am supposed to be studying something that is easily recognizable as clinically relevant. In fact, I feel like I am always doing translational research—I am just *reverse translating* from bedside to bench instead of the other way around. By studying how viruses change their behavior, spread through communities, and make people sick, we end up learning a lot about how evolution works. Since evolution is arguably the framework for all biological sciences, a clear understanding of evolution pays off in many ways.

I became a virologist by accident. I began graduate school convinced that there was nothing more important than cellular signaling. Early on, I went to a seminar where my future mentor, Julie Overbaugh, spoke about a lymphoma-derived feline leukemia virus (FeLV) that had picked up a piece of a cellular gene and made a mutant version of the corresponding protein. Tumors and signaling—this seemed like virology with a purpose. Upon joining Julie’s lab, I quickly realized that the viruses themselves were more interesting. At the time, FeLV was considered a small animal model system for both cancer and AIDS. Most circulating strains weren’t very virulent, but, like other retroviruses, they sometimes acquired the ability to induce mutations in the host or to infect and kill new cell types.

My project was to understand how one such minimally virulent clone evolved to cause tumors in some cats and an AIDS-like immunodeficiency in others. In the first case, we found that the cellular gene contained within the viral genome functioned as a tumor-inducer, or oncogene. By analyzing how the virus evolved to make a rearranged version of an important cellular protein, we, in turn, learned a little bit about how that protein works in normal conditions. In a second study, we identified and characterized the cellular receptor for a class of FeLVs that, like HIV, cause depletion of immune cells. Whereas the tumor-causing FeLVs made a rearranged version of a cellular gene, the AIDS-causing FeLVs entered cells by taking advantage of a protein encoded by a virus-like gene in the cat genome. Both projects began with a simple question: “How did the virus evolve to do this?” In both cases, the virus proved to be an excellent instructor in molecular biology.

When I finished my clinical training and returned to the lab, I felt like it was really time to focus on disease-related research. Still, I found myself drawn to the basic questions. Having studied the end result of viral evolution, I joined Raul Andino’s lab to learn about the process itself. Viruses are wonderful systems for experimental evolution because they evolve over days to weeks as opposed to millennia. Viruses with RNA genomes make mutations up to a thousand times more frequently than DNA viruses and other microbes. Because mutations are the raw materials of evolution, RNA viruses are often described as masters of adaptation. It’s clear, however, that mutation rate isn’t everything, because viruses with the same mutation rate can have very different rates of evolution. At a molecular level, a high mutation rate is definitely a mixed blessing, as very few mutations are beneficial to the virus and most of them are very bad. This led me to wonder whether viruses differed in their tolerance to mutation and whether they had figured out ways to cope with their extremely high mutation rates. We found that the genes of poliovirus are organized in a way that minimizes the negative impact of mutation, possibly while maintaining the virus’ access to the beneficial ones.

In my own laboratory, we’ve begun to study mutational tolerance and the limits to viral evolution in a variety of systems. We’re trying to understand whether influenza virus is more or less sensitive to mutation than poliovirus. Along the way, we’ve found that, like other RNA viruses, influenza virus is exquisitely sensitive to drugs that increase its mutation rate. This was a particularly exciting finding, as other groups had recently shown that favipiravir, a broad-spectrum antiviral in late stage development, might also exert its effect by increasing viral mutation rates.

Our studies of mutation rates have led us to develop a number of methods for describing and measuring viral diversity in human hosts. Viruses make so many mutations that an infected person will harbor a mixture of closely related variants, some of which are transmitted to the next host with each sneeze, pee, or poop. In ongoing collaborations, we are using our methods to learn how viruses evolve within people and how they change when they move from one person to the next. Perhaps my new response at cocktail parties will be, “I study viruses. You’d be surprised how much you can learn about evolution from someone’s bodily fluids!”

**Image 1:**
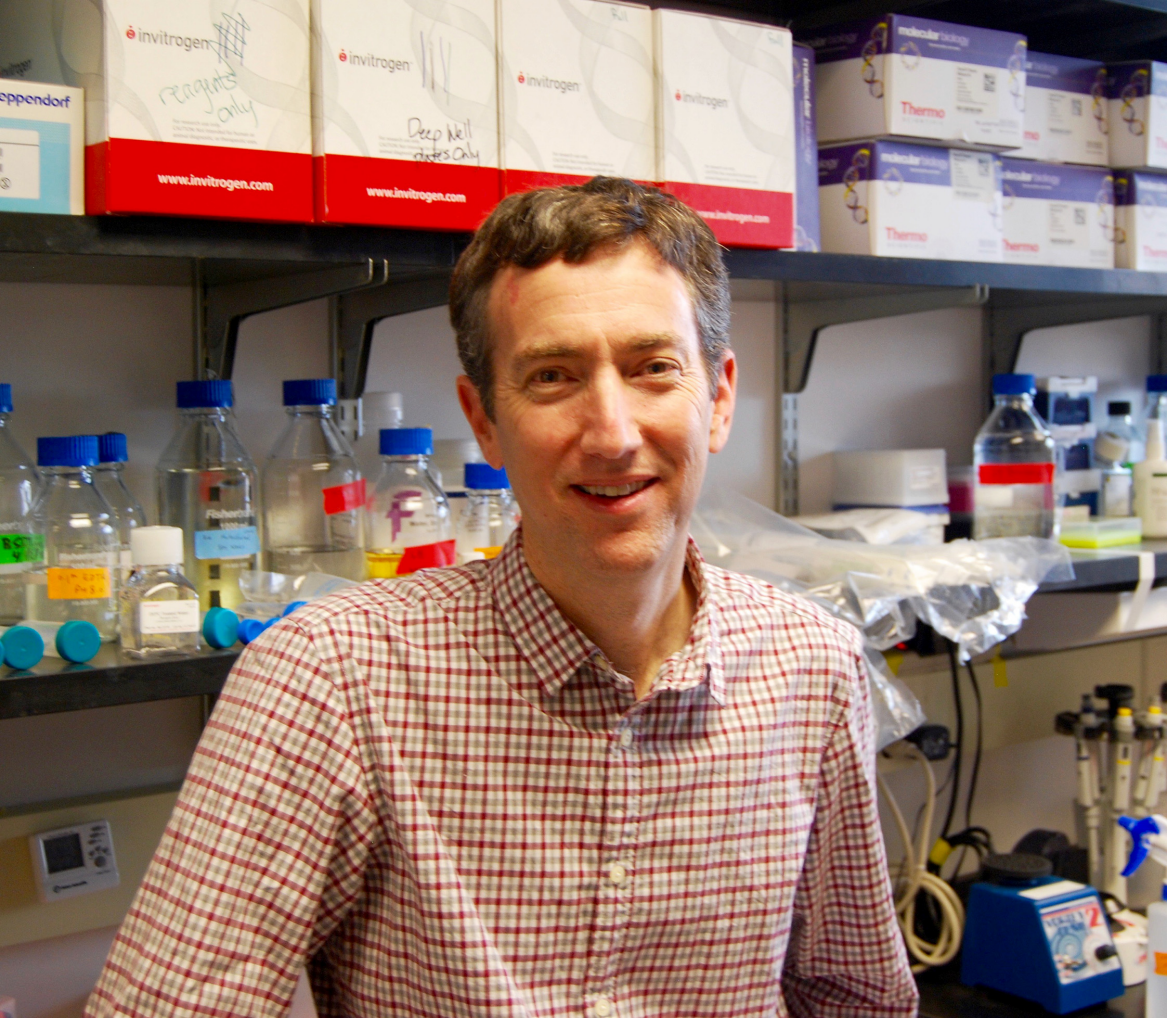
Adam S. Lauring.

